# Supplementary material for: Hierarchically porous and single Zn atom-embedded carbon molecular sieves for H2 separations
Source: Nat Commun. 2024 Jul 7;15:5688. doi: 10.1038/s41467-024-49961-z (PMC11227577; doi:10.1038/s41467-024-49961-z)
Supplement: Supplementary file 1 — Supplementary Information [file 41467_2024_49961_MOESM1_ESM.pdf]

**Supplementary Information**

# Hierarchically porous and single Zn atom-embedded carbon molecular sieves for $H_2$ separations

Leiqing Hu[1], Won Il Lee[2], Soumyabrata Roy[3], Ashwanth Subramanian[2], Kim Kisslinger[4], Lingxiang Zhu[5], Shouhong Fan[6], Sooyeon Hwang[4], Vinh T Bui[1], Thien Tran[1], Gengyi Zhang[1], Yifu Ding[6], Pulickel M. Ajayan[3], Chang-Yong Nam[2,4], and Haiqing Lin[1*]

[1] Department of Chemical and Biological Engineering, University at Buffalo, The State University of New York, Buffalo, NY 14260, USA

[2] Department of Materials Science and Chemical Engineering, Stony Brook University, Stony Brook, NY 11794, USA

[3] Department of Materials Science and NanoEngineering, Rice University, Houston, TX, 77005 USA

[4] Center for Functional Nanomaterials, Brookhaven National Laboratory, Upton, NY 11973, USA

[5] Department of Energy, National Energy Technology Laboratory, Pittsburgh, PA 15236, USA

[6] Department of Mechanical Engineering, University of Colorado, Boulder, CO 80309, USA

* Corresponding author: Haiqing Lin, E-mail: haiqingl@buffalo.edu

**Supplementary Note 1. Morphologies and physical properties of sMMM CMS films**

The composition of the *s*MMM film was obtained using the reported method of mass analysis.[1] Briefly, an *s*MMM sample with a known content of PBI and ZIF-8 (Zn(2-mIm)$_2$) was used for analysis. First, the mass loss was measured after 2-h treatment at 450 °C in a $N_2$ atmosphere, which is ascribed to the loss of 2-mIm from amorphous ZIF-8. Second, the sample was burned in the air at 800 °C, and the mass is the ZnO, which can be used to calculate the total Zn in the sample. Third, the mass of 2-mIm can be calculated by substracting the $Zn^{2+}$ and PBI mass from the total sample mass. Supplementary Table 1 shows that the sMMM contains 9.1 mass% crystalline ZIF-8 and 11 mass% amorphous ZIF-8 with a Zn content of 5.9 mass%. Additionally, the total Zn/2-mIm molar ratio was 0.51, consistent with the literature.[1]

The CMS films were also prepared from PBI/2-mIm blends using the same procedures. The materials exhibit gas transport properties similar to PBI CMS because the 2-mIm evaporates from the film at ~ 200 °C. On the other hand, $Zn(NO_3)_2$ $6H_2O$ could not be dissolved in the PBI solution without the 2-mIm, and thus, we can't prepare homogeneous films. These results confirm the need for both $Zn^{2+}$ and 2-mIm (1:2) in the PBI to form the *s*MMM samples.

**Supplementary Table 1.** Composition of the sMMM sample determined by mass analysis.

| Parameter | Composition (mass%) | | | Zn (mass%) | Molar ratio of Zn to 2-mIm |
|---|---|---|---|---|---|
| | PBI | Amorphous ZIF-8 | Crystalline ZIF-8 | | |
| Value | 79.9 | 11 | 9.1 | 5.9 | 0.51 |

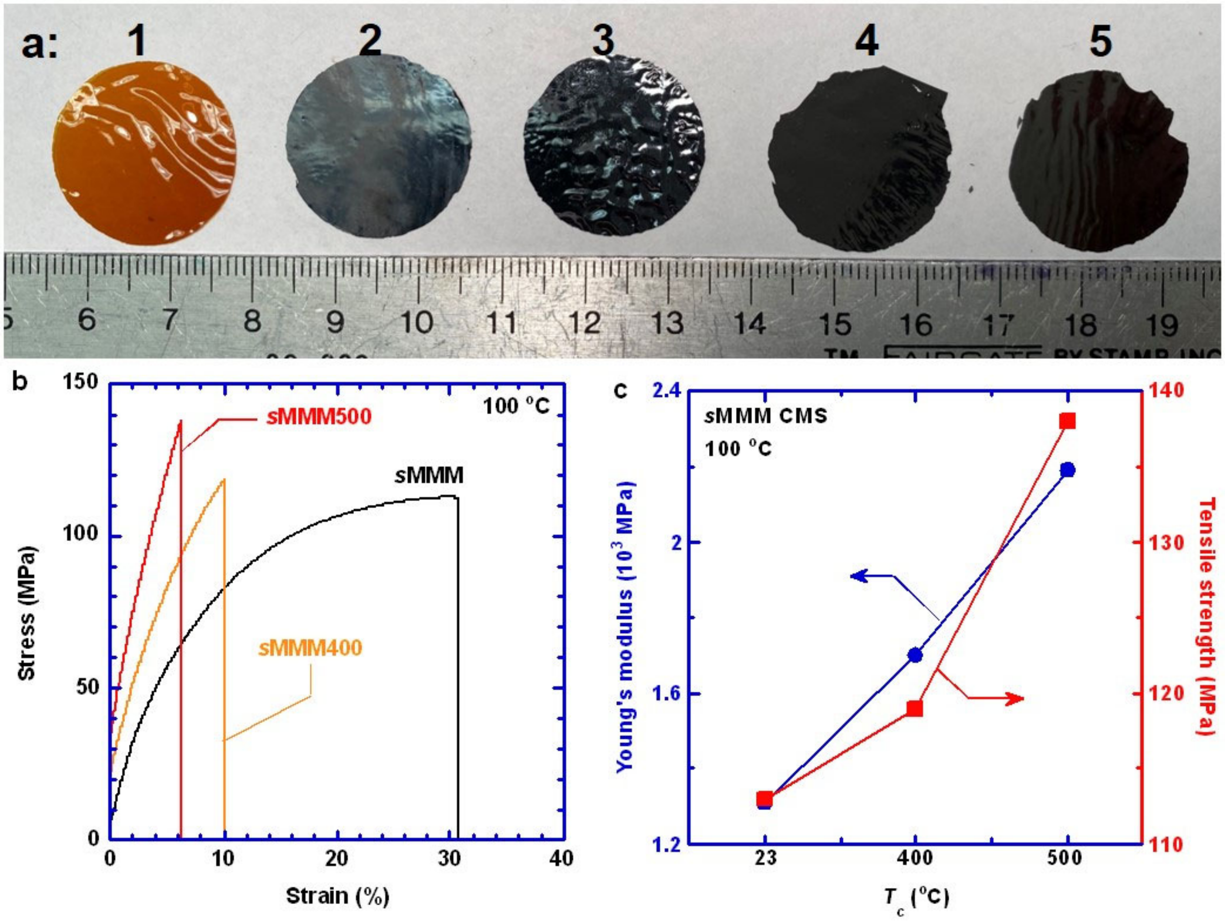


**Supplementary Fig. 1.** (a) Photos of (1) *s*MMM, (2) *s*MMM450, (3) *s*MMM550, (4) *s*MMM700, and (5) *s*MMM900. (b) Tensile stress–strain curves of *s*MMM, *s*MMM450, and *s*MMM500 at 100 °C. (c) Young's modulus and tensile strength of *s*MMM CMS as a function of $T_c$ at 100 °C.

**Supplementary Note 2.** Supplementary Fig. 1a shows that the *s*MMM films change from orange to dark during the carbonization because of the loss of organic groups. Supplementary Fig. 1b shows strain-stress tensile plots of sMMM, *s*MMM450, and *s*MMM500 at 100 °C. The carbonization decreases the fracture strain but increases the modulus and strength value (Supplementary Fig. 1c), indicating that the carbonization leads to a ductile-to-brittle transition. The films were too fragile to measure when $T_c$ exceeded 500 °C. Supplementary Fig. 1d and e show Zn atoms are distributed on both cross-section and surface of sMMM550.

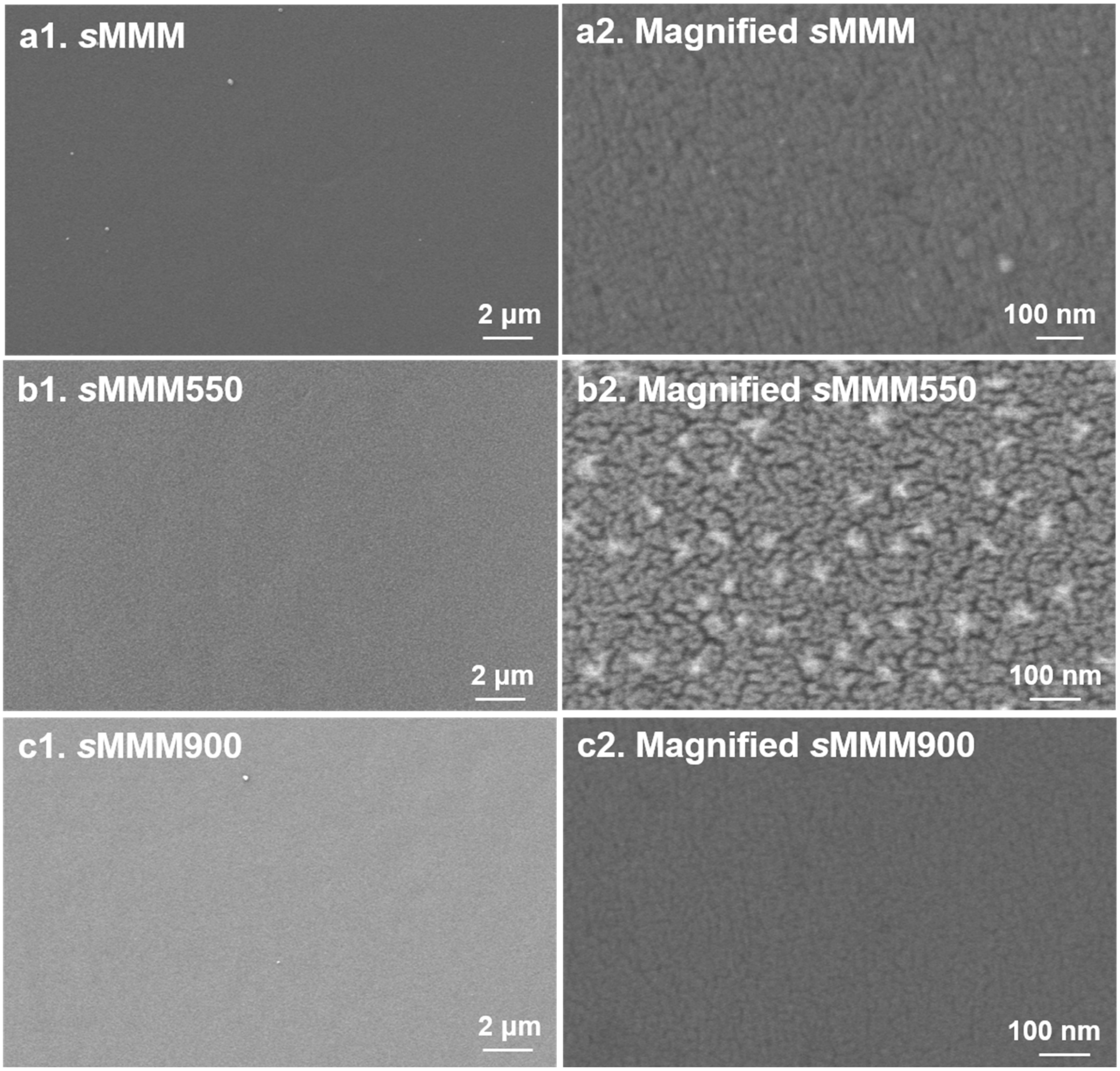


**Supplementary Fig. 2.** SEM surface images: (a) *s*MMM, (b) *s*MMM500, and (c) *s*MMM900.

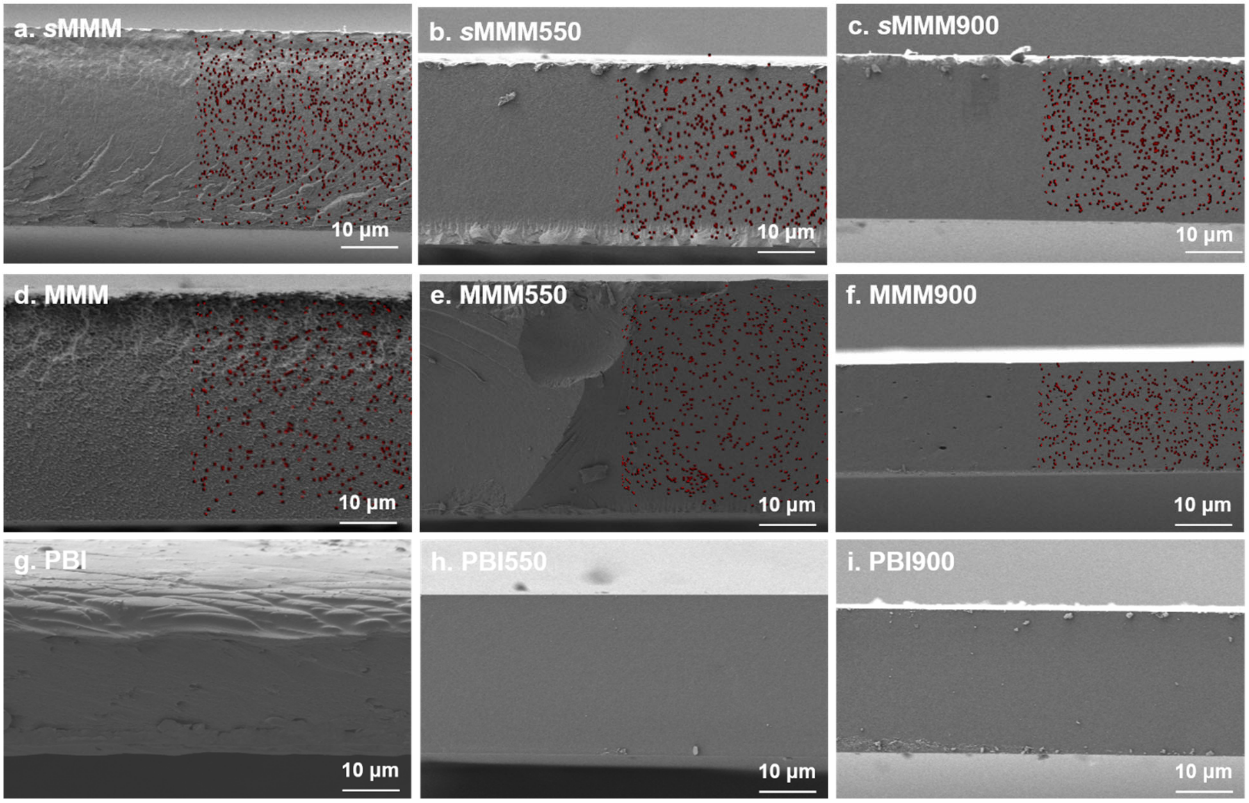


**Supplementary Fig. 3.** SEM cross-sectional images and Zn elemental EDS mappings: (a) *s*MMM, (b) *s*MMM500, (c) *s*MMM900, (d) MMM, (e) MMM500, (f) MMM900, (g) PBI, (h) PBI500, and (i) PBI900. Red spots are Zn elements in a-f.

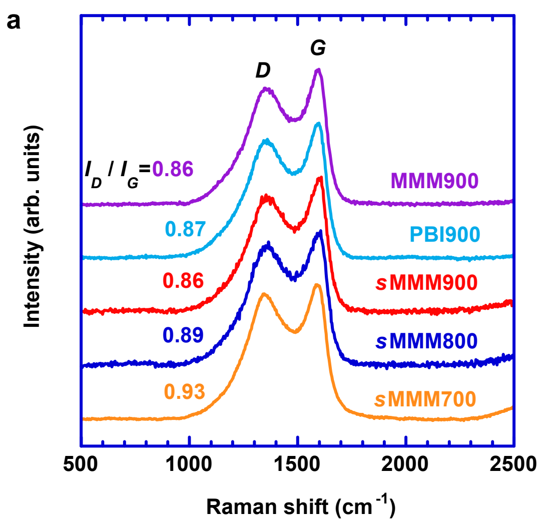


**Supplementary Fig. S4.** (a) Raman spectra of *s*MMM CMS, MMM900, and PBI900. The CMS samples with $T_c$ of 550 °C or below (PBI, MMM or *s*MMM) do not show the carbon peaks. (b) WAXD patterns of MMM, PBI, and their CMS films.

**Supplementary Note 3.** To understand carbon structures in those CMS films, Raman spectra were obtained, as shown in Supplementary Fig. 4. No peak can be detected in CMS films carbonized at 550 °C or below, indicating incomplete carbonization. The D peak at 1340 cm$^{-1}$ (representing the disordered carbon) and G peak at 1600 cm$^{-1}$ (representing the oriented graphitic carbon) can be observed in CMS films carbonized at 700 − 900 °C. Increasing $T_c$ decreases the $I_D/I_G$ ratio value, consistent with the increasing graphitic N peak in *s*MMM900 and the graphitic plane from WAXD patterns.[2] Interestingly, PBI900 and MMM900 exhibit $I_D/I_G$ ratio values similar to *s*MMM900, indicating that ZIF-8 has a negligible effect on the evolution of carbon structures at $T_c$ = 900 °C.

Supplementary Fig. 4b shows WAXD patterns of MMM, PBI, and their CMS films. The sharp peaks of ZIF-8 in MMM films disappear in MMM CMS films, indicating its crystalline structure becomes amorphous after the carbonization. Generally, *d*-spacing decreases with increasing $T_c$ for all CMS samples.

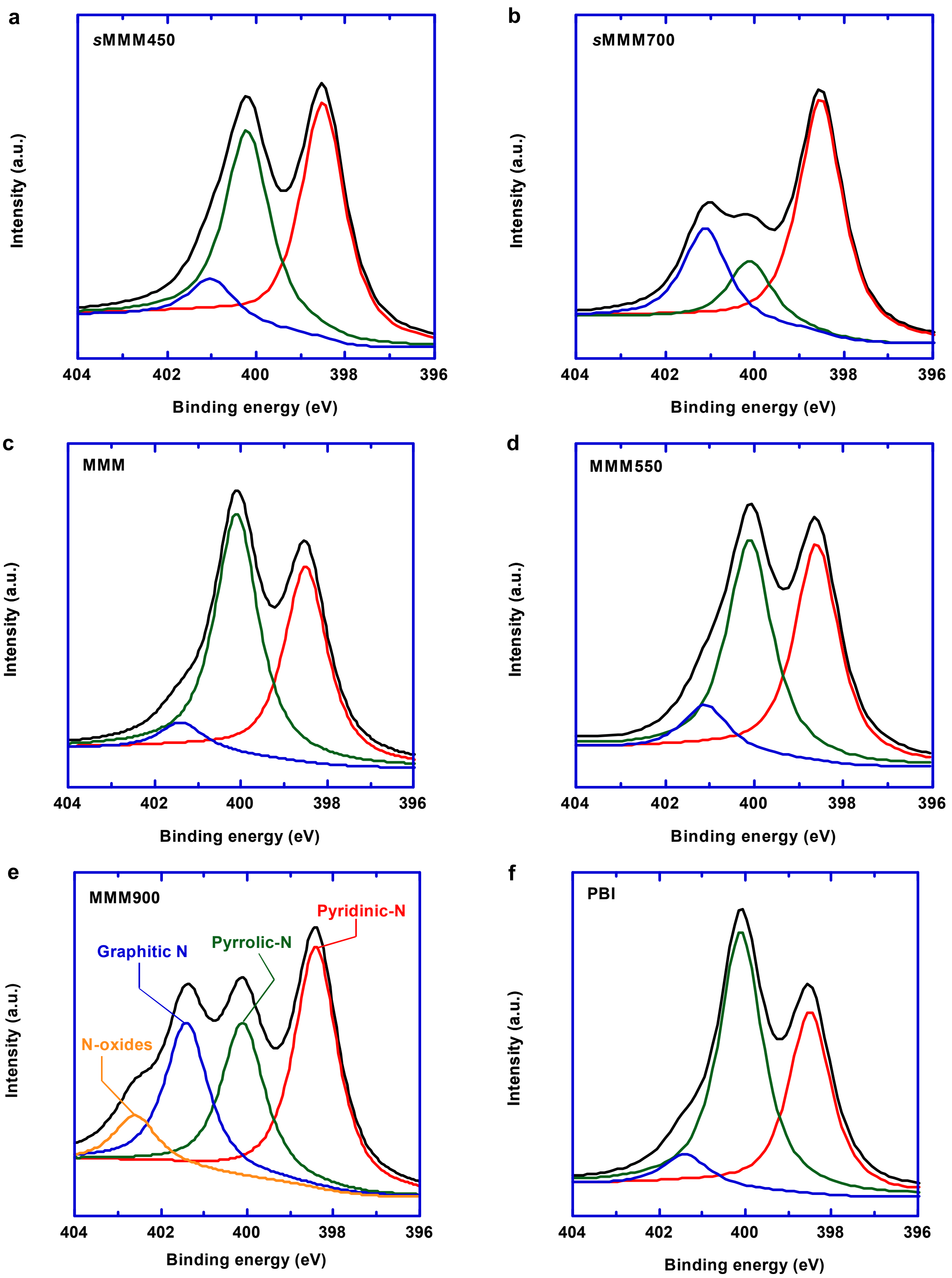
a
sMMM450
b
sMMM700
c
MMM
d
MMM550
e
MMM900
Pyridinic-N
Pyrrolic-N
Graphitic N
N-oxides
f
PBI
Intensity (a.u.)
Binding energy (eV)
404
402
400
398
396

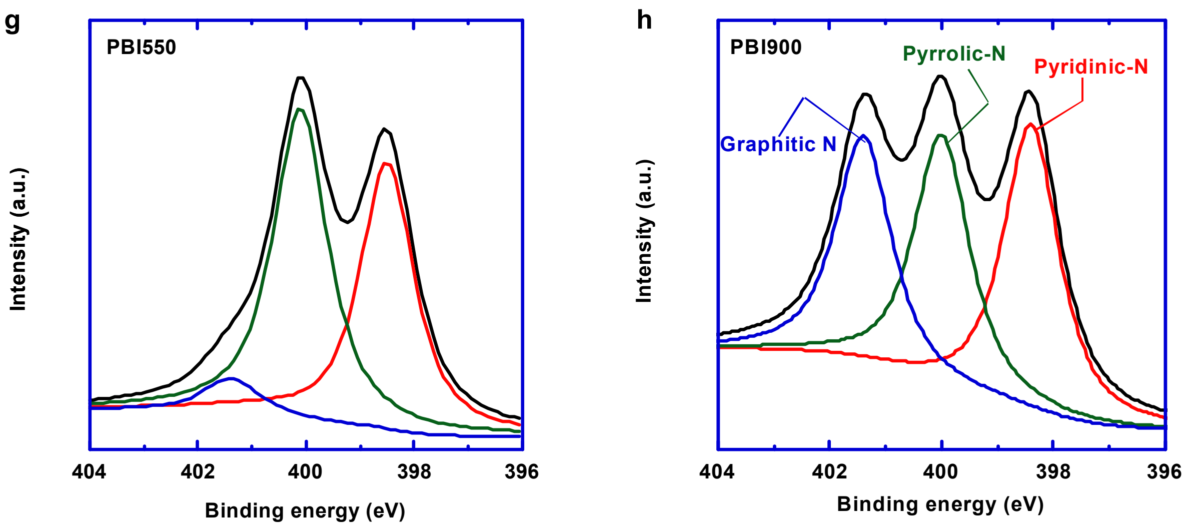


**Supplementary Fig. 5.** N*1s* XPS spectra of film samples.

**Supplementary Note 4.** Increasing $T_c$ increases the mass loss from the precursors by carbonization (Supplementary Fig. 6a). In *s*MMM, the order of thermal stabilities of three components is as follows: amorphous ZIF-8 < crystalline ZIF-8 < PBI.[1] Therefore, for *s*MMM550, the mass loss mainly happens at the ramping step due to the degradation of the amorphous ZIF-8. Likewise, *s*MMM CMS films exhibit higher mass losses than MMM CMS and PBI CMS (Supplementary Fig. 6a). Additionally, ZIF-8 has ~ 20% mass loss when being carbonized at 550 °C due to the release of methyl groups on 2-mIm. Thus, MMM CMS films show higher mass loss than PBI CMS due to the faster degradation of ZIF-8 than PBI. Another potential reason for greater mass losses in *s*MMM CMS and MMM CMS is the catalyzed effect of the Zn metal on the polymer decomposition.[3] Moreover, the mass loss of *s*MMM450 is 8.7%, corresponding to the content (7.9 %) of 2-mIm in the *s*MMM.

Supplementary Fig. 6b-e shows $CO_2$ and $N_2$ sorption isotherms of those CMS films at 0 °C and −196 °C, respectively. The pore volume and surface area increase with increasing carbonization temperature. Besides, Supplementary Fig. 6i,j exhibits an increased porosity with increasing $T_c$, consistent with the mass loss. The *s*MMM CMS films have higher $\rho_s$ and $\rho_b$ values than PBI CMS and MMM CMS (Supplementary Table 2).

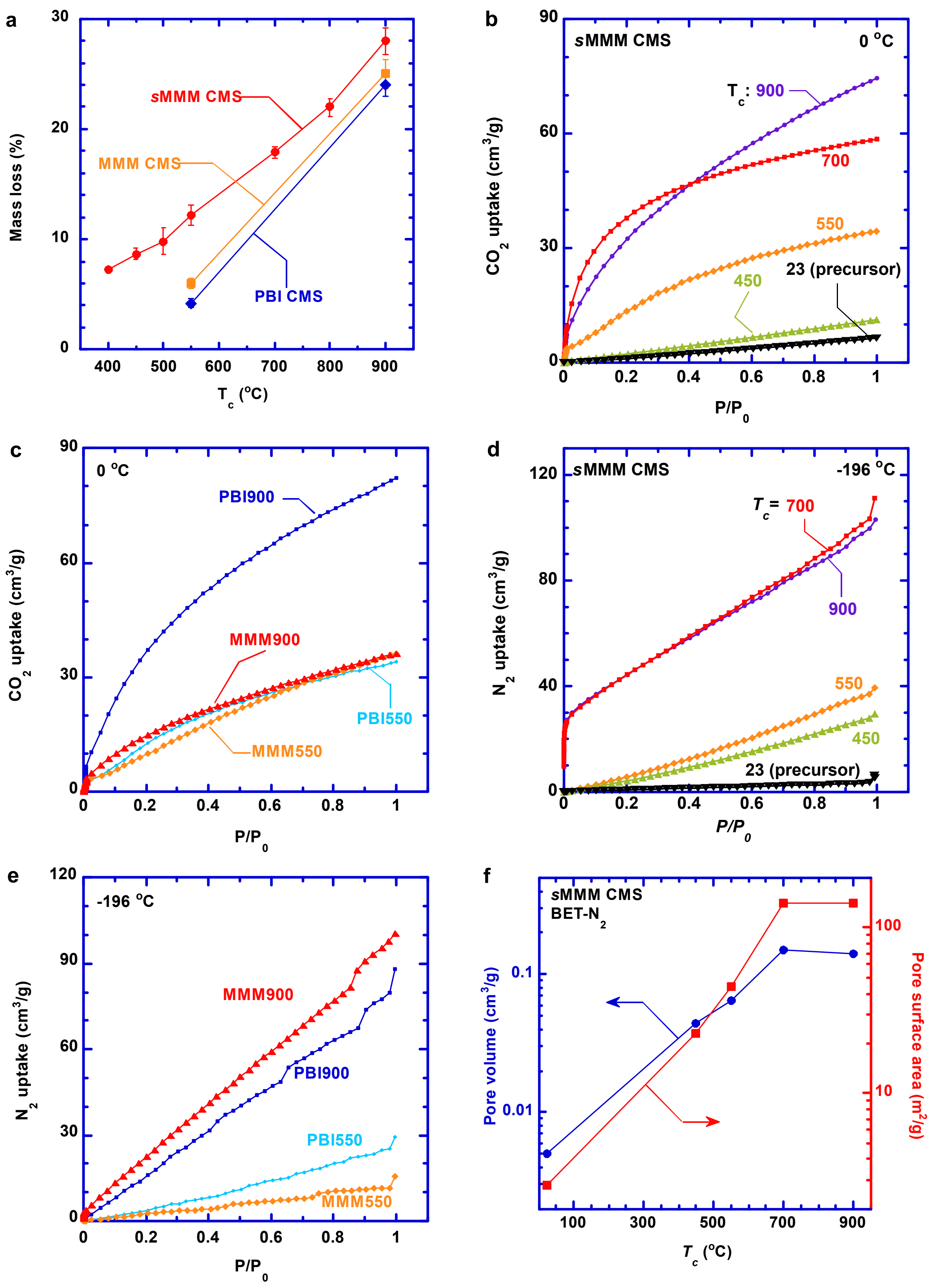
a
Mass loss (%)
sMMM CMS
MMM CMS
PBI CMS
$T_c$ (°C)
b
sMMM CMS
0 °C
$T_c$: 900
700
550
450
23 (precursor)
$CO_2$ uptake ($cm^3/g$)
$P/P_0$
c
0 °C
PBI900
MMM900
PBI550
MMM550
$CO_2$ uptake ($cm^3/g$)
$P/P_0$
d
sMMM CMS
-196 °C
$T_c$ = 700
900
550
450
23 (precursor)
$N_2$ uptake ($cm^3/g$)
$P/P_0$
e
-196 °C
MMM900
PBI900
PBI550
MMM550
$N_2$ uptake ($cm^3/g$)
$P/P_0$
f
sMMM CMS
BET-$N_2$
Pore volume ($cm^3/g$)
Pore surface area ($m^2/g$)
$T_c$ (°C)

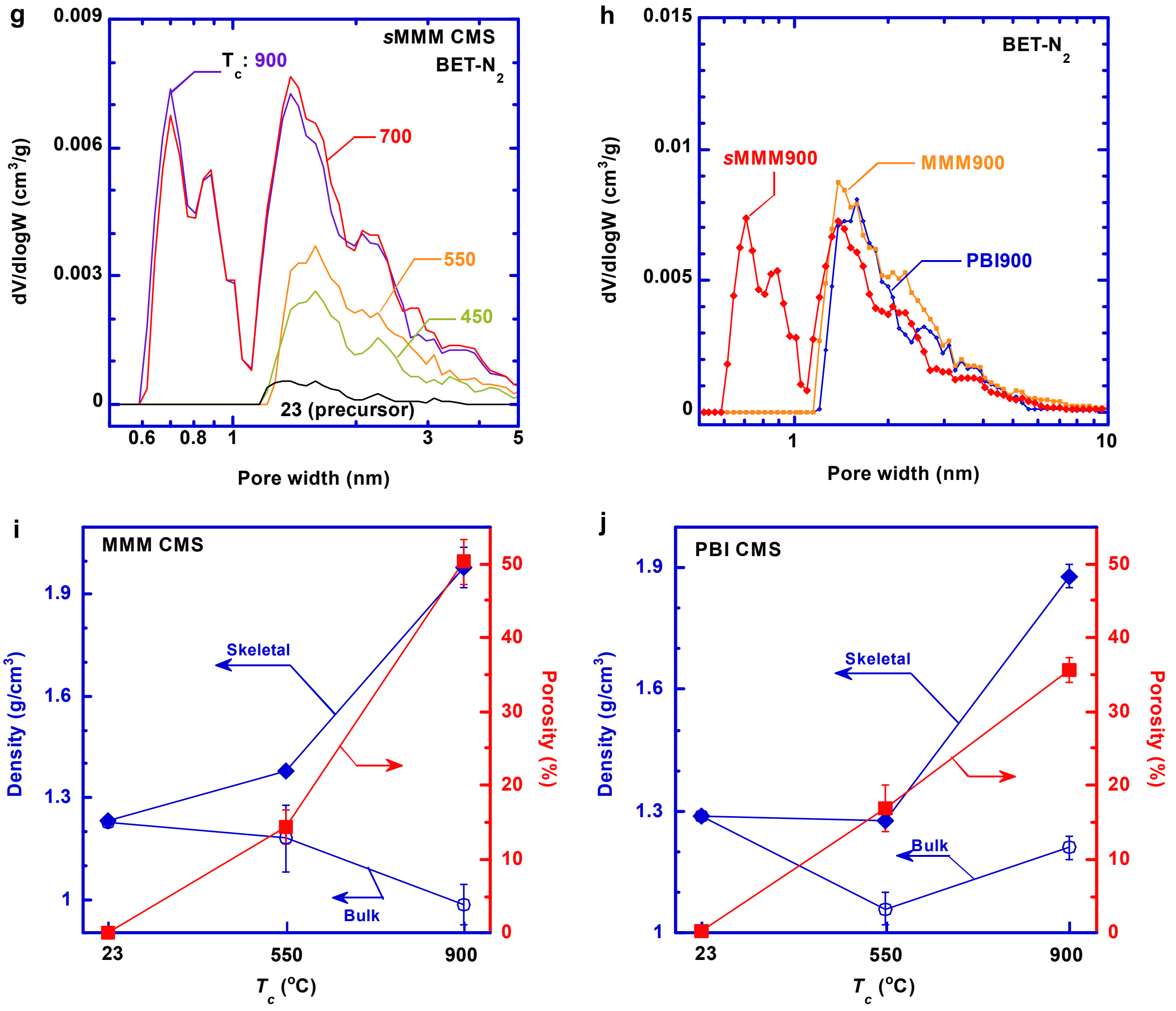


**Supplementary Fig. 6.** (a) Mass loss of all CMS films after carbonization. $CO_2$ sorption isotherms of (b) *s*MMM CMS and (c) MMM CMS and PBI CMS at 0 °C. $N_2$ sorption isotherms of (d) *s*MMM CMS and (e) MMM CMS and PBI CMS at −196 °C. (f) Pore volume and surface area, and (g) pore size distributions of sMMM CMS films, and (h) all CMS900 films calculated by non-local density functional theory (NLDFT) from $N_2$ adsorption at −196 °C. Skeletal density, bulk density, and porosity of (i) MMM CMS and (j) PBI CMS films. Error bars in Figure a,i, and j are estimated mean absolute errors

**Supplementary Table 2.** Skeletal and bulk densities, porosity, *d*-spacing, pore volume, and surface area of all samples.

| Samples | Skeletal density ($g/cm^3$) | Bulk density ($g/cm^3$) | Porosity (%) | *d*-spacing (Å) | BET $CO_2$ | | BET $N_2$ | |
|---|---|---|---|---|---|---|---|---|
| | | | | | Pore volume ($cm^3/g$) | Surface area ($m^2/g$) | Pore volume ($cm^3/g$) | Surface area ($m^2/g$) |
| *s*MMM | 1.353 ± 0.008 | 1.325 ± 0.005 | 2.1 ± 0.4 | 3.5 | 0.009 | 11 | 0.005 | 2.8 |
| *s*MMM400 | 1.306 ± 0.008 | 1.160 ± 0.020 | 11 ± 2 | 4.9 | N/A† | N/A | N/A | N/A |
| *s*MMM450 | 1.283 ± 0.013 | 1.120 ± 0.029 | 13 ± 2 | N/A | 0.016 | 17 | 0.044 | 23 |
| *s*MMM500 | 1.344 ± 0.030 | 1.162 ± 0.018 | 14 ± 2 | 4.5 | N/A | N/A | N/A | N/A |
| *s*MMM550 | 1.441 ± 0.010 | 1.187 ± 0.038 | 18 ± 3 | 4.1 | 0.049 | 43 | 0.064 | 44 |
| *s*MMM700 | 1.753 ± 0.013 | 1.351 ± 0.020 | 23 ± 1 | 3.7 | 0.083 | 68 | 0.15 | 140 |
| *s*MMM800 | 1.885 ± 0.016 | 1.461 ± 0.011 | 22 ± 1 | 3.6 | N/A | N/A | N/A | N/A |
| *s*MMM900 | 1.994 ± 0.018 | 1.346 ± 0.009 | 33 ± 1 | 3.6 | 0.11 | 88 | 0.14 | 140 |
| PBI | 1.289 ± 0.002 | 1.287 ± 0.004 | 0.2 ± 0.1 | 4.0 | N/A | N/A | N/A | N/A |
| PBI550 | 1.275 ± 0.010 | 1.060 ± 0.031 | 17 ± 3 | 4.3 | 0.049 | 37 | 0.037 | 16 |
| PBI900 | 1.878 ± 0.028 | 1.210 ± 0.028 | 36 ± 2 | 3.8 | 0.12 | 86 | 0.11 | 50 |
| MMM | 1.230 ± 0.008 | 1.229 ± 0.006 | 0.1± 0.1 | N/A | N/A | N/A | N/A | N/A |
| MMM550 | 1.379 ± 0.011 | 1.180 ± 0.011 | 14 ± 1 | 4.5 | 0.051 | 49 | 0.019 | 9.2 |
| MMM900 | 1.980 ± 0.059 | 0.985 ± 0.023 | 50 ± 3 | 3.7 | 0.052 | 43 | 0.14 | 61 |

Note: † N/A means data is not available.

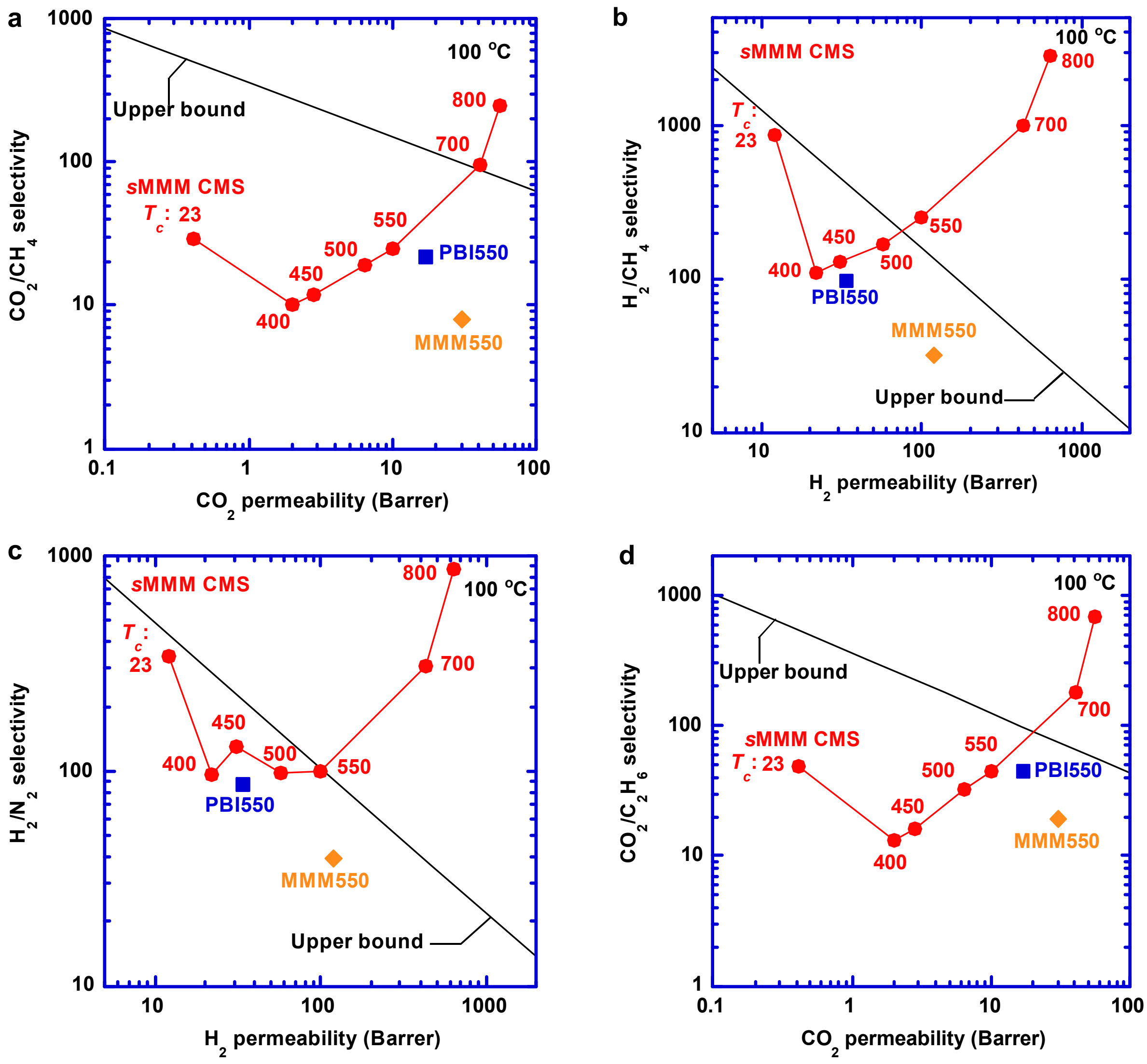


**Supplementary Fig. 7.** (a) $CO_2/CH_4$, (b) $H_2/CH_4$, (c) $H_2/N_2$, and (d) $CO_2/C_2H_6$ separation performance of all CMS samples versus Robeson's upper bounds at 100 °C.

**Supplementary Note 4.** $H_2$ sorption is too low to measure, and $C_2H_6$ can serve as a surrogate for $H_2$ since both do not have polar or quadrupole moments to exhibit specific interactions with the CMS materials. Gas sorption ($C_A$) in glassy polymer and CMS membranes can be described using the dual-mode sorption model as expressed using Supplementary Equation 1:[4]

$$C_A = k_D p_A + \frac{C'_H b p_A}{1 + b p_A} \qquad \text{(Supplementary Eq. 1)}$$

where $k_D$ is Henry's constant, $C'_H$ is Langmuir sorption capacity, and $b$ is the affinity parameter. As shown in Figure S8, both $CO_2$ and $C_2H_6$ sorption in all samples can be satisfactorily described using the dual-mode sorption model with the fitting parameter values listed in Supplementary

Table 4. Carbonization increases $CO_2$ sorption and the corresponding $k_D$ and $C'_H$ values. Interestingly, $C'_H$ value decreases when $T_c$ increases to 900 °C, indicating the formation of the cut-off at 3.3 Å. Supplementary Table 5 summarizes $CO_2$ and $C_2H_6$ diffusivity and solubility in *s*MMM CMS, PBI550, and MMM550 films at 8 atm. For *s*MMM CMS, both $CO_2$ and $C_2H_6$ solubility increase with increasing $T_c$ due to the increased porosity, and $CO_2/C_2H_6$ solubility selectivity decreases. When $T_c$ reaches 900 °C, $C_2H_6$ solubility decreases and $CO_2/C_2H_6$ solubility selectivity increases because of the shrunk ultramicropores that become inaccessible for the sorption of larger $C_2H_6$ molecules. MMM550 and PBI550 show $CO_2$ and $C_2H_6$ solubility similar to *s*MMM550, indicating in-situ carbonized ZIF-8 has similar gas solubility to carbonized PBI at 550 °C. Both $CO_2$ and $C_2H_6$ diffusivity of *s*MMM CMS increase first due to the increased porosity before decreasing resulting from the shrinkage of ultramicropores. Particularly, $C_2H_6$ and $CO_2$ diffusivity reach the highest values when $T_c$ is 400 and 700 °C, respectively, consistent with the gradually decreased ultramicropores. Likewise, $CO_2/C_2H_6$ diffusivity selectivity increases with increasing $T_c$. Particularly, $CO_2/C_2H_6$ diffusivity selectivity reaches 150 when $T_c$ is 900 °C. Compared to MMM550 and PBI550, *s*MMM550 has lower $CO_2$ and $C_2H_6$ diffusivity, which can be attributed to the tighter structure of its precursor.

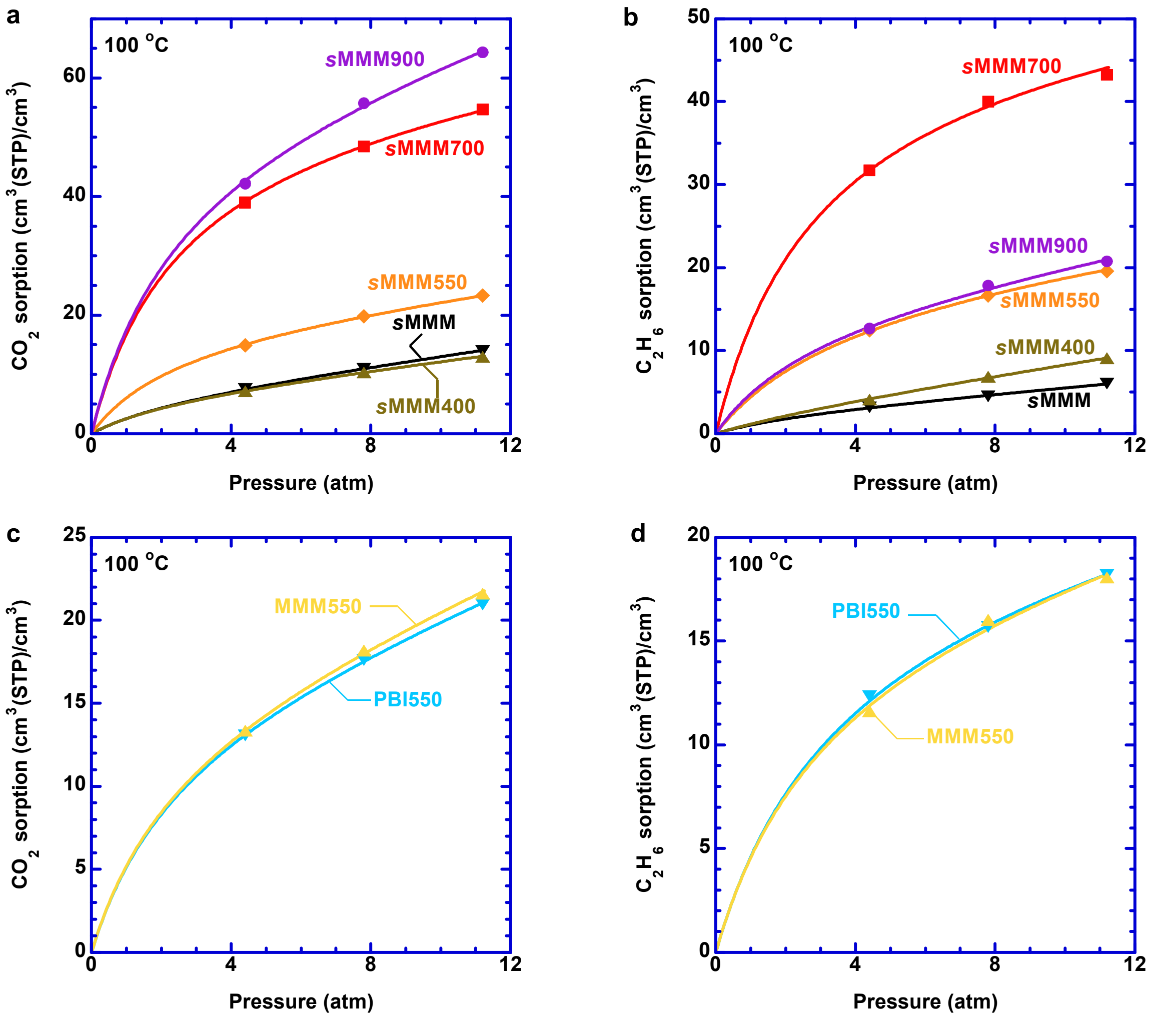


**Supplementary Fig. 8.** (a) $CO_2$ and (b) $C_2H_6$ sorption isotherms of sMMM and sMMM CMS at 100 °C. (c) $CO_2$ and (d) $C_2H_6$ sorption isotherms of MMM550 and PBI550 at 100 °C.

**Supplementary Table 3.** Pure-gas permeability and selectivity of PBI, MMM, and their CMS films at 100 °C and 8 atm.

| Sample | Gas permeability (Barrer) | | | | | Gas selectivity | | | | |
|---|---|---|---|---|---|---|---|---|---|---|
| | $H_2$ | $CO_2$ | $N_2$ | $CH_4$ | $C_2H_6$ | $H_2/CO_2$ | $H_2/N_2$ | $H_2/CH_4$ | $H_2/C_2H_6$ | $CO_2/CH_4$ |
| PBI | 9.0 | 0.53 | -- | -- | -- | 17 | -- | -- | -- | -- |
| PBI550 | 80 | 17 | 0.92 | 0.82 | 0.38 | 4.7 | 87 | 98 | 210 | 21 |
| PBI900 | 57 | 2.6 | -- | -- | -- | 22 | -- | -- | -- | -- |
| MMM | 30 | 3.7 | 0.14 | 0.30 | 0.080 | 8.1 | 210 | 100 | 380 | 12 |
| MMM550 | 120 | 30 | 3.1 | 3.8 | 1.6 | 4 | 39 | 32 | 75 | 8.0 |
| MMM900 | 86 | 3.0 | -- | -- | -- | 29 | -- | -- | -- | -- |

**Supplementary Table 4.** Parameters of the dual-mode sorption model for $CO_2$ and $C_2H_6$ sorption in sMMM, sMMM CMS, MMM550, and PBI550 at 100 °C. The units for $k_D$, $b$, and $C'_H$ are $cm^3(STP)\ cm^{-3}\cdot atm^{-1}$, $atm^{-1}$, and $cm^3(STP)\ cm^{-3}$, respectively.

| Samples | $CO_2$ | | | $C_2H_6$ | | |
|---|---|---|---|---|---|---|
| | $k_D$ | $b$ | $C'_H$ | $k_D$ | $b$ | $C'_H$ |
| *s*MMM | 0.80 | 0.38 | 6.3 | 0.36 | 0.39 | 2.4 |
| *s*MMM400 | 0.70 | 0.40 | 6.4 | 0.65 | 0.25 | 2.5 |
| *s*MMM550 | 0.69 | 0.39 | 19 | 0.50 | 0.26 | 19 |
| *s*MMM700 | 0.74 | 0.40 | 56 | 0.40 | 0.34 | 50 |
| *s*MMM900 | 1.8 | 0.41 | 54 | 0.74 | 0.34 | 16 |
| PBI550 | 0.78 | 0.41 | 15 | 0.33 | 0.29 | 19 |
| MMM550 | 0.84 | 0.41 | 15 | 0.36 | 0.32 | 18 |

**Supplementary Table 5.** $CO_2$ diffusivity and solubility, $C_2H_6$ solubility and solubility, $CO_2/C_2H_6$ diffusivity selectivity, and $CO_2/C_2H_6$ solubility selectivity of *s*MMM550, PBI550, and MMM550 at 100 °C and 8 atm.

| Samples | Gas diffusivity ($\times10^{-8}$ $cm^2/s$) | | Gas solubility ($cm^3(STP)/(cm^3$ atm)) | | $CO_2/C_2H_6$ diffusivity selectivity | $CO_2/C_2H_6$ solubility selectivity |
|---|---|---|---|---|---|---|
| | $CO_2$ | $C_2H_6$ | $CO_2$ | $C_2H_6$ | | |
| *s*MMM | 0.22 | 0.011 | 1.4 | 0.58 | 20 | 2.4 |
| *s*MMM400 | 1.1 | 0.13 | 1.3 | 0.88 | 8.8 | 1.5 |
| *s*MMM550 | 3.0 | 0.078 | 2.5 | 2.1 | 38 | 1.2 |
| *s*MMM700 | 5.0 | 0.034 | 6.2 | 5.1 | 150 | 1.2 |
| *s*MMM900 | 0.088 | -- | 7.2 | 2.3 | -- | 3.1 |
| PBI550 | 5.6 | 0.14 | 2.3 | 2.1 | 41 | 1.1 |
| MMM550 | 9.9 | 0.58 | 2.3 | 2.1 | 17 | 1.1 |

**Supplementary Table 6.** $P_{A,0}$ and $E_{P,A}$ values for $N_2$, $CH_4$, and $C_2H_6$ permeation in *s*MMM550.

| Gas type | $E_{P,A}$ (kJ/mol) | $P_{A,0}$ (×10$^3$ Barrer) |
|---|---|---|
| $N_2$ | 10 ± 1 | 0.028 |
| $CH_4$ | 15 ± 1 | 0.053 |
| $C_2H_6$ | 19 ± 1 | 0.15 |

**Supplementary Table 7.** Comparison of the $P_{A,0}$ and $E_{P,A}$ values for $H_2$ and $CO_2$ permeation in all samples.

| Samples | $E_{P,A}$ (kJ/mol) | | $P_{A,0}$ (×10$^3$ Barrer) | |
|---|---|---|---|---|
| | $H_2$ | $CO_2$ | $H_2$ | $CO_2$ |
| *s*MMM | 19 ± 1 | 20 ± 1 | 6.2 | 0.24 |
| *s*MMM550 | 11 ± 1 | 7.2 ± 1 | 3.1 | 0.10 |
| *s*MMM900 | 20 ± 1 | 34 ± 2 | 38 | 41 |
| *s*MMM900[1] | 25 ± 2 | 36 ± 3 | 140 | 80 |
| PBI | 21 ± 1 | 22 ± 2 | 8.9 | 1.4 |
| PBI550 | 15 ± 1 | 12 ± 4 | 12 | 0.79 |
| PBI900 | 21 ± 1 | 18 ± 1 | 41 | 0.80 |
| MMM | 20 ± 3 | 16 ± 1 | 16 | 0.44 |
| MMM550 | 17 ± 1 | 15 ± 3 | 46 | 4.2 |
| MMM900 | 19 ± 1 | 30 ± 1 | 51 | 46 |

Note: [1] mixed-gas data.

**Supplementary Note 5.** The temperature effect on gas permeability of all samples can be described using the Arrhenius equation.[5]

$$P_A = P_{0,A} \exp\left(-E_{p,A}/RT\right) \qquad \text{(Supplementary Eq. 2)}$$

where $R$ and $T$ are the gas constant and testing temperature, respectively. $P_{0,A}$ and $E_{P,A}$ are the pre-exponential factor and activation energy of the permeation, respectively, and their values are listed in Supplementary Tables 6 and 7. In *s*MMM550 film, $N_2$ has the lowest $E_{P,A}$ value due to its smaller molecular size (Supplementary Table 6), while $C_2H_6$ has the largest molecular size and thus the highest $E_{P,A}$ value.

In all three types of CMS films, $E_{P,A}$ values for both $H_2$ and $CO_2$ permeation decrease first with increasing $T_c$ because of the increased porosity and before decreasing because of the densification of carbon porous structures.

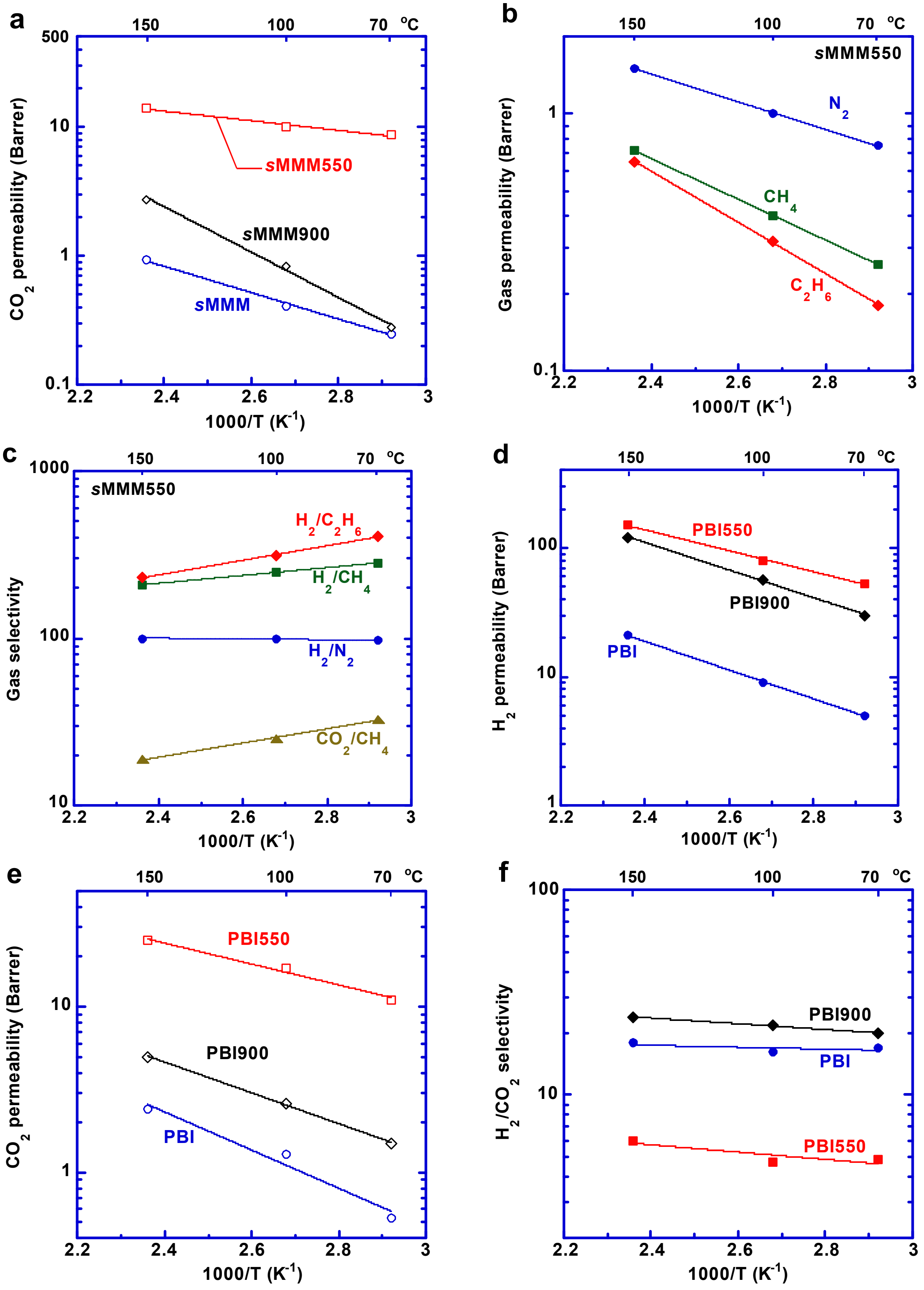

a
b
c
d
e
f
1000/T (K-1)
°C
CO2 permeability (Barrer)
sMMM550
sMMM900
sMMM
Gas permeability (Barrer)
N2
CH4
C2H6
Gas selectivity
H2/C2H6
H2/CH4
H2/N2
CO2/CH4
H2 permeability (Barrer)
PBI550
PBI900
PBI
H2/CO2 selectivity

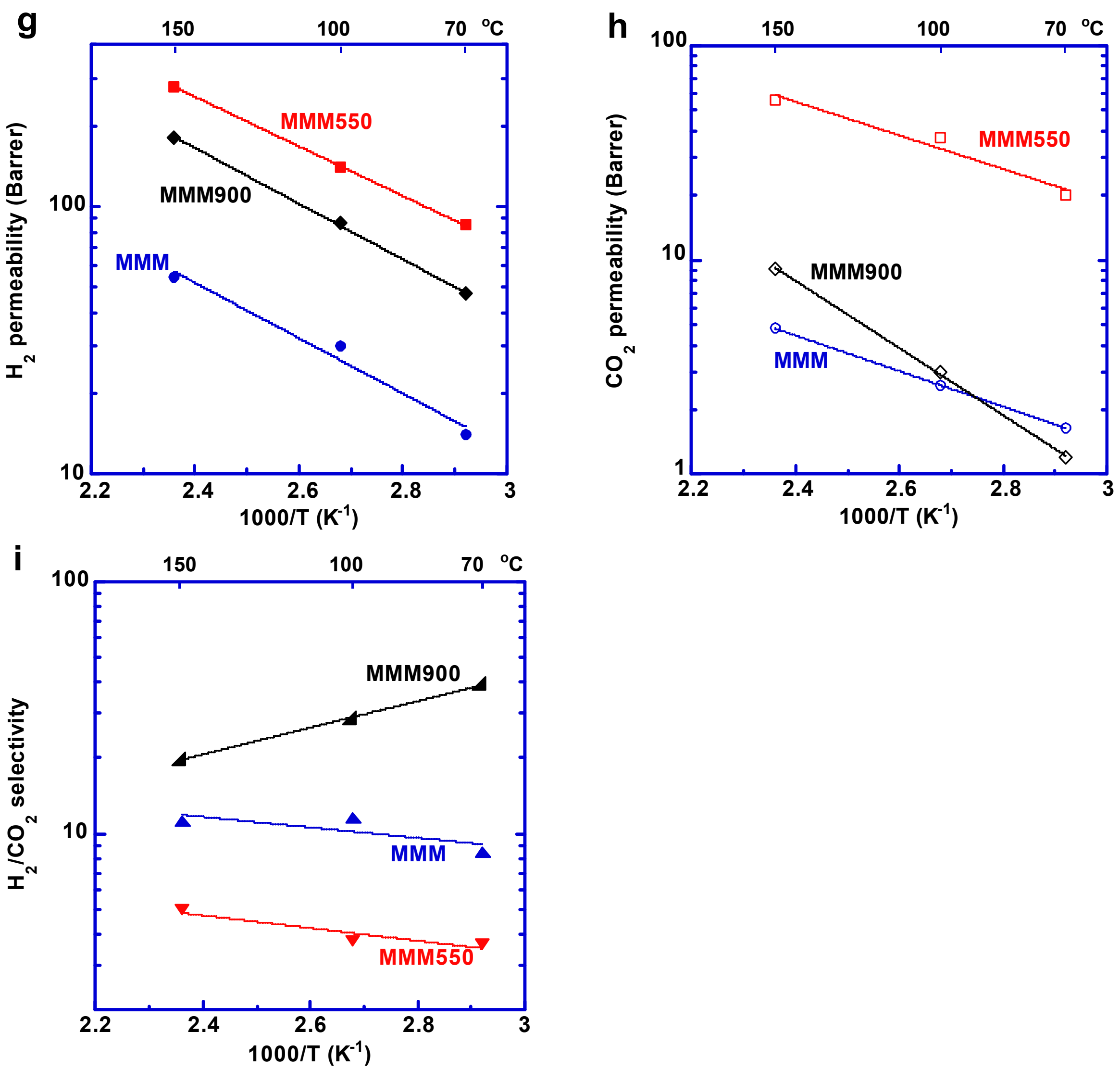


**Supplementary Fig. 9.** Temperature effect on (a) $CO_2$ permeability of *s*MMM and *s*MMM CMS films, (b) $N_2$, $CH_4$ and $C_2H_6$ permeability of *s*MMM550 film, (c) gas selectivity of *s*MMM550 film, (d) $H_2$ and (e) $CO_2$ permeability, and (f) $H_2/CO_2$ selectivity of PBI and PBI CMS films, and (g) $H_2$ permeability, (h) $CO_2$ permeability, and (i) $H_2/CO_2$ selectivity of MMMs and MMM CMS films.

**Supplementary Table 8.** $H_2/CO_2$ separation properties in selected membrane materials for comparison (Fig. 5e). The gas mixtures contained 50% $CO_2$ and 50% $H_2$.

| Materials | | | Mixed- or pure- gas | Temp. (°C) | $H_2$ perm. (Barrer) | $H_2/CO_2$ selectivity | Ref. |
|---|---|---|---|---|---|---|---|
| CMS in this study | 1 | *s*MMM900 | Pure | 70 | 37 | 130 | This study |
| | 2 | | Pure | 100 | 66 | 80 | |
| | | | Mixed | | 55 | 61 | |
| Leading CMS materials | 3 | PBI-PPA/600 | Pure | 150 | 140 | 58 | 5 |
| | | | Mixed | 150 | 110 | 31 | |
| | 4 | PBI/900 | Pure | 100 | 55 | 80 | 6 |
| | | | Mixed | 100 | 36 | 53 | |
| | 5 | Cellulose/700 | Pure | 130 | 773 | 49.5 | 2 |
| | | | Mixed | 90 | 225 | 31 | |
| | 6 | Kapton/1000 | Pure | 50 | 7.2 | 160 | 7 |
| | 7 | PBI-Matrimid/1000 | Pure | 35 | 94 | 24 | 8 |
| | 8 | Cellophane/600 | Pure | 30 | 39 | 59 | 9 |
| | 9 | PABZ-6FDA/850 | Pure | 35 | 190 | 13 | 10 |
| | 10 | Polyimide/700 | Pure | 100 | 180 | 10 | 11 |
| | 11 | MTI aramide/925 | Pure | 35 | 9.1 | 366 | 12 |
| | | | Mixed | 35 | 3.5 | 156 | |
| | 12 | PAI/925 | Pure | 35 | 33 | 94 | 13 |
| | | | Mixed | 35 | 16 | 57 | |
| | 13 | CANAL-TB/900 | Pure | 35 | 4.95 | 248 | 14 |
| | 14 | | Mixed | 100 | 8.2 | 174 | |
| State-of-the-art polymers | 15 | PBI-$(H_3PO_4)_{0.16}$ | Mixed | 150 | 12 | 34 | 15 |
| | 16 | PBI-TCL-6 h | Mixed | 150 | 19 | 18 | 16 |
| | 17 | PBI-$TMA_{0.22}$ | Mixed | 150 | 16 | 28 | 17 |
| | 18 | IP BILPs | Mixed | 150 | 13 | 32 | 18 |
| | 19 | SCA4/PBI-17 | Mixed | 150 | 16 | 45 | 19 |
| | 20 | SCA8/PBI-10 | Mixed | 150 | 37 | 23 | 20 |
| | 21 | Pd/PBI-58 | Mixed | 175 | 43 | 31 | 21 |
| | 22 | Pd/PBI-23 | Pure | 60 | 176 | 10 | 22 |
| | 23 | ZIF-8/PBI-30 | Mixed | 35 | 105 | 10 | 23 |
| | 24 | ZIF-7/PBI-50 | Mixed | 120 | 200 | 9 | 24 |
| | 25 | CPAM-15 | Mixed | 200 | 50 | 24 | 1 |

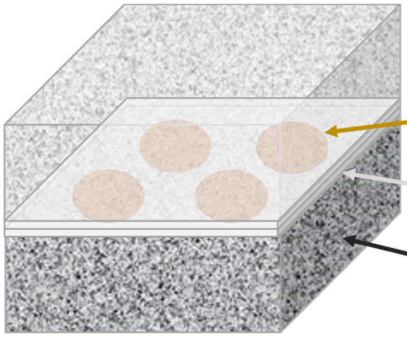


**Supplementary Fig. 10.** The schematic of the sandwiched precursor polymeric films for carbonization.

**Supplementary References**


1 Hu, L. *et al.* In Situ Growth of Crystalline and Polymer-Incorporated Amorphous ZIFs in Polybenzimidazole Achieving Hierarchical Nanostructures for Carbon Capture. *Small* **18**, 2201982, (2022).

2 Lei, L. *et al.* Carbon hollow fiber membranes for a molecular sieve with precise-cutoff ultramicropores for superior hydrogen separation. *Nat. Commun.* **12**, 268, (2021).

3 Hu, L. *et al.* Palladium-Percolated Networks Enabled by Low Loadings of Branched Nanorods For Enhanced $H_2$ Separations. *Adv. Mater.* **35**, 2301007, (2023).

4 Hu, L. *et al.* Supramolecular polymer networks of ion-coordinated polybenzimidazole with simultaneously improved $H_2$ permeability and $H_2/CO_2$ selectivity. *Macromolecules* **55**, 6901-6910, (2022).

5 Hu, L. *et al.* Tailoring Sub-3.3 Å Ultramicropores in Advanced Carbon Molecular Sieve Membranes for Blue Hydrogen Production. *Sci. Adv.* **8**, eabl8160, (2022).

6 Omidvar, M. *et al.* Unexpectedly Strong Size-Sieving Ability in Carbonized Polybenzimidazole for Membrane $H_2/CO_2$ Separation. *ACS Appl. Mater. Interfaces* **11**, 47365-47372, (2019).

7 Hatori, H., Takagi, H. & Yamada, Y. Gas separation properties of molecular sieving carbon membranes with nanopore channels. *Carbon* **42**, 1169-1173, (2004).

8 Hosseini, S. S. & Chung, T. S. Carbon membranes from blends of PBI and polyimides for $N_2/CH_4$ and $CO_2/CH_4$ separation and hydrogen purification. *J. Membr. Sci.* **328**, 174-185, (2009).

9 Campo, M. C., Magalhaes, F. D. & Mendes, A. Carbon molecular sieve membranes from cellophane paper. *J. Membr. Sci.* **350**, 180-188, (2010).

10 Liang, J. *et al.* Effects on carbon molecular sieve membrane properties for a precursor polyimide with simultaneous flatness and contortion in the repeat unit. *ChemSusChem* **13**, 5531-5538, (2020).

11 Ngamou, P. T., Ivanova, M., Guillon, O. & Meulenberg, W. A. High-performance carbon molecular sieve membranes for hydrogen purification and pervaporation dehydration of organic solvents. *J. Mater. Chem. A* **7**, 7082-7091, (2019).

12 Iyer, G. M. & Zhang, C. Precise Hydrogen Sieving by Carbon Molecular Sieve Membranes Derived from Solution-Processable Aromatic Polyamides. *ACS Mater. Lett.* **5**, 243-248, (2022).

13 Iyer, G. M., Ku, C.-E. & Zhang, C. Polyamide-imide copolymer-derived carbon molecular sieve membranes for efficient hydrogen/carbon dioxide separation. *Carbon* **216**, 118598, (2024).

14 Hazazi, K. *et al.* Catalytic arene-norbornene annulation (CANAL) ladder polymer derived carbon membranes with unparalleled hydrogen/carbon dioxide size-sieving capability. *J. Membr. Sci.* **654**, 120548, (2022).

15 Zhu, L., Swihart, M. T. & Lin, H. Unprecedented Size-Sieving Ability in Polybenzimidazole Doped with Polyprotic Acids for Membrane $H_2/CO_2$ Separation. *Energy Environ. Sci.* **11**, 94-100, (2018).

16 Zhu, L., Swihart, M. T. & Lin, H. Tightening Polybenzimidazole (PBI) Nanostructure via Chemical Cross-Linking for Membrane $H_2/CO_2$ Separation. *J. Mater. Chem. A* **5**, 19914-19923, (2017).

17 Hu, L. *et al.* Supramolecular Assemblies of Polybenzimidazole and Aromatic Polycarboxylic Acids with Superior Mechanical and $H_2/CO_2$ Separation Properties. *J. Mater. Chem. A* **10**, 10872-10879, (2022).

18 Shan, M. *et al.* Facile Manufacture of Porous Organic Framework Membranes for Precombustion $CO_2$ Capture. *Sci. Adv.* **4**, eaau1698, (2018).

19 Wu, J. & Chung, T. Supramolecular Polymer Network Membranes with Molecular-Sieving Nanocavities for Efficient Pre-Combustion $CO_2$ Capture. *Small Methods* **6**, 2101288, (2021).

20 Wu, J., Liang, C., Naderi, A. & Chung, T. Tunable Supramolecular Cavities Molecularly Homogenized in Polymer Membranes for Ultraefficient Precombustion $CO_2$ Capture. *Adv. Mater.* **34**, 2105156, (2021).

21 Zhu, L. *et al.* Sorption-Enhanced Mixed Matrix Membranes with Facilitated Hydrogen Transport for Hydrogen Purification and $CO_2$ Capture. *Adv. Funct. Mater.* **29**, 1904357, (2019).

22 Villalobos, L. F., Hilke, R., Akhtar, F. H. & Peinemann, K. V. Fabrication of Polybenzimidazole/Palladium Nanoparticles Hollow Fiber Membranes for Hydrogen Purification. *Adv. Energy Mater.* **8**, 1701567, (2018).

23 Yang, T., Shi, G. & Chung, T. S. Symmetric and Asymmetric Zeolitic Imidazolate Frameworks (ZIFs)/Polybenzimidazole (PBI) Nanocomposite Membranes for Hydrogen Purification at High Temperatures. *Adv. Energy Mater.* **2**, 1358-1367, (2012).

24 Yang, T., Xiao, Y. & Chung, T. S. Poly-/metal-benzimidazole nano-composite membranes for hydrogen purification. *Energy Environ. Sci.* **4**, 4171-4180, (2011).
